# Glucose metabolism perturbations influence tumor microenvironments via LINC01139 pathway and facilitate immunotherapy in hepatocellular carcinoma

**DOI:** 10.1016/j.gendis.2024.101302

**Published:** 2024-04-08

**Authors:** Yueying Gao, Jinyang Yu, Zhi Li, Kefan Liu, Jiwei Pan, Ya Zhang, Yanlin Ma, Jiwei Zhang, Zhigang Liu, Yongsheng Li

**Affiliations:** a Hainan Provincial Key Laboratory for Human Reproductive Medicine and Genetic Research, Department of Reproductive Medicine, Hainan Provincial Clinical Research Center for Thalassemia, Key Laboratory of Reproductive Health Diseases Research and Translation, Ministry of Education, The First Affiliated Hospital of Hainan Medical University, College of Biomedical Information and Engineering, Hainan Medical University, Haikou, Hainan 571101, China; b School of Interdisciplinary Medicine and Engineering, Harbin Medical University, Harbin, Heilongjiang 150081, China; c The MOE Key Laboratory for Standardization of Chinese Medicines, Institute of Chinese Materia Medica, Shanghai University of Traditional Chinese Medicine, Shanghai 201203, China; d Affiliated Foshan Maternity & Child Healthcare Hospital, Southern Medical University, Guangzhou, Guangdong 510000, China

Hepatocellular carcinoma (HCC) is one of the most common tumors with high prevalence and death rate, which lacks effective targeting and immunotherapy currently. Metabolic reprogramming, which provides an inherent advantage for tumor cells to compete for nutrition, plays important roles in the development and progression of cancers.^1^ Increasing studies indicated that metabolic reprogramming of tumors can remodel the microenvironment through different signaling pathways and thus promote the development of tumors.^2^^,^^3^ In addition, long non-coding RNAs (lncRNAs) represent a subgroup of noncoding RNAs and have been shown to play extensive regulatory functions in cancer, such as signaling and immune pathways. Besides, it has been widely clarified that lncRNAs play important roles in bridging metabolic reprogramming and immunology.^4^ However, knowledge regarding the lncRNAs perturbed by metabolic reprogramming in HCC is still lacking. Therefore, an in-depth understanding of the downstream lncRNA pathways of metabolic reprogramming is of great significance for identifying new therapeutic targets for HCC.

Glucose metabolic reprogramming, known as the Warburg effect, has been identified as one of the metabolic hallmarks of tumor cells. We first collected 67 glucose metabolism-related genes and investigated the genetic alterations (including somatic mutations and copy number variations) in HCC (Table S1). We found widespread genetic perturbations in glucose metabolism-related genes, and all 67 genes had genetic alterations (somatic mutations and copy number variation) in about 95.25% (361/379) HCC patients (Fig. S1A, 2). PKLR had the highest alteration frequency, in particular the prevalent copy number amplification (Fig. S1A, 3), which has been identified as a regulator of metabolism and targets for cancer treatment.

We next determined to what extent the perturbations of glucose metabolism correlated with clinical significance. We first divided all HCC patients into two groups by considering whether there was genetic alteration (somatic mutations, homozygous deletion, and deep amplification) in glucose metabolism-related genes (altered *vs*. unaltered). We found that there was no significant difference in clinical significance between the two groups of HCC patients, including sex, metastasis, lymph node state, disease and tumor stages, and ethnicity (Fig. S4). However, patients in the altered groups were with significantly worse disease-free survival (Fig. S1B; log-rank *P* = 0.029). Thus, we next investigated whether there were some differences in the molecular characteristics between the two groups. Hypoxia has become an organizational principle of cell evolution, metabolism, and pathology. We found that patients with glucose metabolism-related genes altered have significantly higher hypoxia scores than those without alterations (Fig. S1C; *P* = 0.0052). The fractions of genome altered and the tumor mutation burden in the patients of altered groups were significantly higher than those in the unaltered groups (Fig. S1D, E; *P* = 0.035, *P* = 5.2E-6, respectively).

The interaction between the immune system and metabolism has been demonstrated to play important roles in cancer. We next investigated the immune cell infiltrations of HCC patients and found that patients in two groups were with significantly differences in the levels of immune cell infiltration (Fig. S5; supplemental materials and methods). Patients in the altered groups were likely to have more T cell infiltrations than those in the unaltered groups (Fig. S5, 6). In addition, we found that several immune checkpoint genes were significantly highly expressed in the altered patients (Fig. S5, 6), such as CD276, CD80, and CD27. These results suggest that patients in the altered groups were likely to respond to immunotherapy. We also investigated the cancer pathway activities and identified five cancer pathways with significant activities between two groups of patients (Fig. S1F, G). In particular, the PI3K-AKT-MTOR signaling pathway exhibited significantly higher activities in the altered group (Fig. S1G; *P* < 0.01). Moreover, several genes (*e.g.*, CDK1, PLCB1, and HRAS) in this pathway exhibited significantly higher expression in the altered groups (Fig. S1H). These results suggested that the genetic alterations of glucose metabolism perturbed the tumor microenvironment and cancer signaling pathways to promote cancer development.

We next investigated to what extent the lncRNA transcriptomes were perturbed by the alterations of glucose metabolism in HCC. In total, we identified 82 lncRNAs exhibiting a significant difference in expression between two groups of HCC patients (Fig. 1A; false discovery rate <0.05), including 79 up-regulated and 3 down-regulated lncRNAs. We found that the expression of these lncRNAs was significantly correlated with immune cell infiltrations, expression of immune checkpoints, and cancer pathway activities in HCC (Fig. 1B). Next, we ranked the differentially expressed lncRNAs according to the number of correlated immune and cancer-related features. We found that several cancer-related lncRNAs were ranked top in our list (Table S2), such as SAMMSON, FEZF1-AS1, and LINC-ROR. In particular, we focused on LINC01139 which ranked 7th for further analyses.

**Figure 1:**
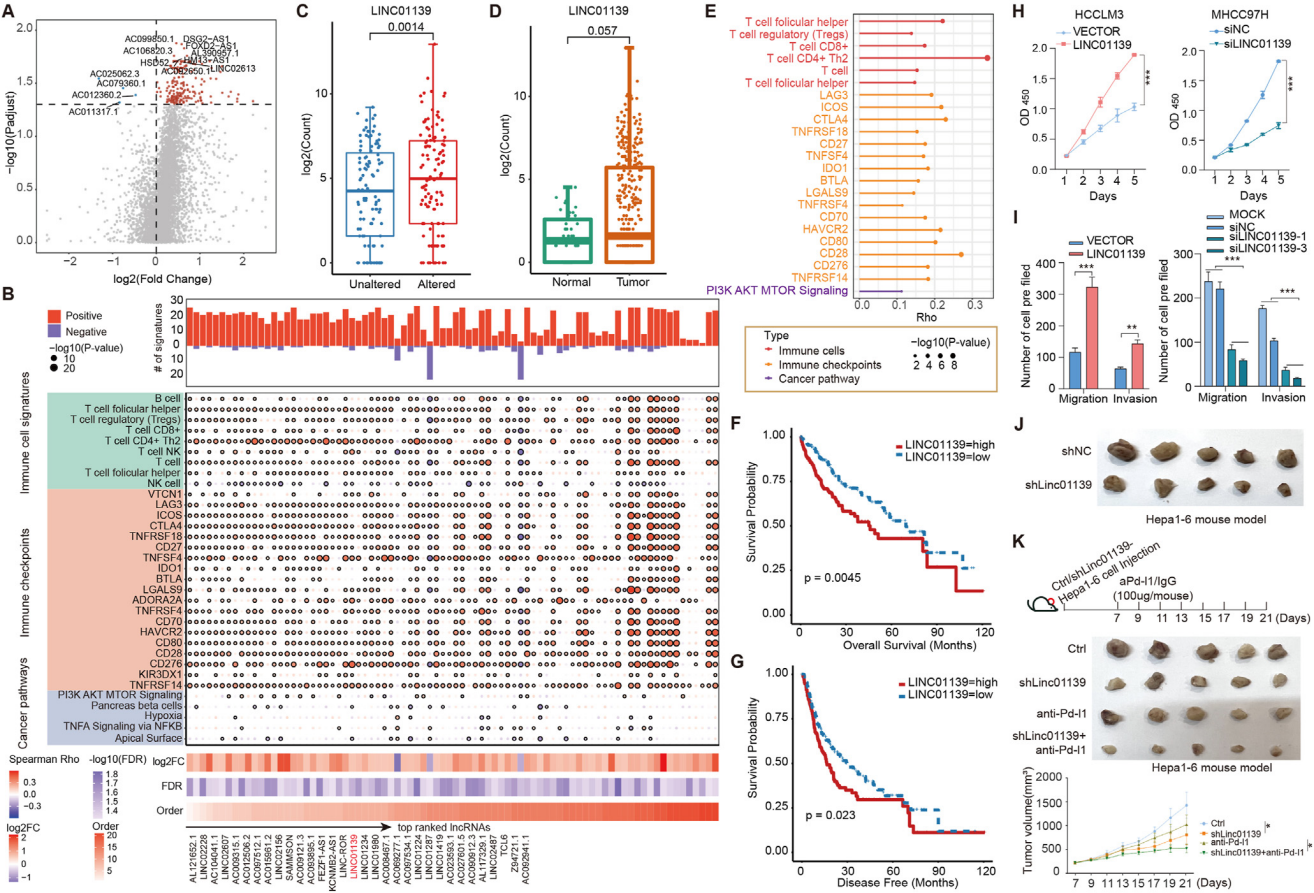
Glucose metabolism perturbations altered oncogenic LINC01139 and silencing oncogenic LINC01139 enhanced the clinical benefits of immunotherapy. **(A)** The volcano plot showing the alterations of lncRNAs perturbed by alterations of glucose metabolism. **(B)** Correlations between expression of lncRNAs and cancer/immune-related signatures. The upper bar plots show the number of signatures correlated with each lncRNA. The middle dot plots show the correlations and significances. The bottom heat maps show the fold changes, false discovery rate, and ranks of lncRNAs. **(C, D)** The boxplots showing the expression of LINC01139 (C) for unaltered *vs*. altered and (D) for normal and cancer. **(E)** The lollipop plot showing the correlations between expression of LINC01139 and cancer/immune-related signatures. **(F, G)** Kaplan–Meier overall and disease-free survival curves. Patients were divided into two groups based on the median expression of LINC01139 (F) for overall survival and (G) for disease-free survival. **(H)** The line graphs showing the effects of overexpression and knockdown of LINC01139 on cell growth. **(I)** The bar plots showing the effects of overexpression and knockdown of LINC01139 on migration and invasion. **(J)** The representative images depicting the tumor size of linc01139 knockdown in mice. **(K)** Rational combination therapy of linc01139 and aPD-L1 in hepatocellular carcinoma. The top panel shows the mouse model used. The middle panel shows representative images of tumors in mice with different treatments. The bottom panel shows the line graphs of tumor volumes.

We found that LINC01139 exhibited significantly higher expression in patients with glucose metabolism perturbations (Fig. 1C; *P* = 0.0014). In addition, LINC01139 was highly expressed in HCC compared with normal controls (Fig. 1D; *P* = 0.057). We found that the expression of LINC01139 was significantly positively correlated with immune and cancer-related features (Fig. 1E), such as T cell infiltrations, expression of CD276, and activities of the PI3K-AKT-MTOR pathway. In particular, LINC01139 was significantly positively co-expressed with several genes (*e.g.*, PLCB2, CDK2, and CDK1), involved in the PI3K-AKT-MTOR signaling pathway (Fig. S7). Survival analysis revealed that HCC patients with high LINC01139 expression had worse overall survival (Fig. 1F; log-rank *P* = 0.0045) and disease-free survival (Fig. 1G; log-rank *P* = 0.023). All these results suggest that perturbations of glucose metabolism significantly altered the lncRNA transcriptome; thus, we prioritized an ontogenetic lncRNA for further development of cancer immunotherapy.

To further investigate the possible function of LINC01139 in HCC pathogenesis, we next performed functional assays in cell lines and mouse models. We overexpressed LINC01139 in HCCLM3 cell line and knocked down the expression of LINC01139 in MHCC97H cell line (Fig. S8). We found colony formation, migration, and invasion were increased in the presence of LINC01139 overexpression (Fig. 1H, I; Fig. S9A). In contrast, we found that LINC01139 knockdown significantly decreased cell growth, migration, and invasion as compared with control (Fig. 1H, I; Fig. S9B). Furthermore, we examined the effect of LINC01139 knockdown on tumor growth *in vivo* using mouse subcutaneous xenograft models. We found that tumor xenografts derived from linc01139-knockdown cells exhibited smaller volumes and lower weights than those from empty vector-transduced cells (Fig. 1J; Fig. S9C, D; *P* < 0.05, *P* < 0.001, respectively). Collectively, these data indicated that the silence of LINC01139 repressed HCC cell growth, invasion, and metastasis.

It is critical to discover additional lncRNA targets for improving the sensitivity of cancer immunotherapy. We found that the expression of LINC01139 was correlated with numerous immune-related signatures (Fig. 1E), suggesting rational combination immunotherapy of LINC01139 in HCC. To further investigate the function of LINC01139 in immunotherapy, we used the Hepa1-6 cell injection mouse model. The mice were treated with sh-linc01139 and Pd-l1 blockade or IgG after HCC implantation (Fig. 1K). We found that the silence of linc01139 and Pd-l1 blockade could inhibit tumor growth (Fig. 1K) and reduce the tumor weight (Fig. S9E; *P* < 0.05). Moreover, the mice treated with the combination of sh-linc01139 and aPd-l1 had a much smaller tumor volume and weight than the other mice (Fig. 1K; Fig. S9E). Overall, these results demonstrated that LINC01139 serves as a potential target to enhance the benefits of immunotherapy in HCC.

In conclusion, our computational and experiment results revealed that glucose metabolism perturbations influence tumor microenvironments and widely alter the lncRNA transcriptomes in HCC. Our findings also suggested that silencing an oncogenic LINC01139 may be a novel synergistic strategy for enhancing HCC immunotherapy sensitivity.

## Ethics declaration

Patient data we used were acquired by publicly available datasets that were collected with patients' informed consent. The mouse model study protocol conformed to the ethical guidelines delineated within the Declaration of Helsinki and its later amendments and was approved by the Institutional Review Boards of Shanghai Immunocan Biotechnology (YMNK-AUA-00601).

## Author contributions

Y.L., Z.L., J.Z., and Y.M. designed this study. Y.G., J.Y., and Z.L. analyzed the data and interpreted the results. K.L., J.P., and Y.Z. performed the experiments and Y.G. performed the data analysis. Y.L., Z.L., J.Z., and Y.M. wrote and edited the manuscript. All authors read and approved the manuscript.

## Funding

This work was supported by the 10.13039/100014717National Natural Science Foundation of China (No. 32322020, 32170676, 31970646, 32060152), the 10.13039/501100005046Natural Science Foundation of Heilongjiang Province, China (Key Program, No. ZD2023C007), Heilongjiang Touyan Innovation Team Program (China), the specific research fund of The Innovation Platform for Academicians of Hainan Province, China, and the project supported by Hainan Province Clinical Medical Center (China).

## Conflict of interests

The authors declared no competing interests.
