# Supplementary material for: Glucose metabolism perturbations influence tumor microenvironments via LINC01139 pathway and facilitate immunotherapy in hepatocellular carcinoma
Source: Genes Dis. 2024 Apr 8;12(2):101302. doi: 10.1016/j.gendis.2024.101302 (PMC11605351; doi:10.1016/j.gendis.2024.101302)
Supplement: Multimedia component 1 [file mmc1.docx]

**Supplemental Materials and methods**

**Cell lines**

The HCC cell lines MHCC97H and HCCLM3 were purchased from American Type Culture Collection (ATCC) and cultured following the recommended instructions. These cells were characterized by Genewiz Inc. (China) using short tandem repeat (STR) markers and were confirmed to be Mycoplasma-free (last tested in year 2017).

**Overexpression and knockdown of LINC01139 in cell lines**

The LINC01139 sequence was synthesized by GENEray Biotechnology (China) and cloned into the eukaryotic expression vector pcDNA3.1 and the lentivirus expression vector pWPXL. The siRNAs of LINC01139 were purchased from RiboBio (China). The validated siRNA sequence of LINC01139 was synthesized and cloned into the siRNA cloning and expression lentivector. The shRNA sequences were as follows:

siRNA#1：UGGAUUUGUACCAUUCUUCUG

siRNA#2：ACUCAUUGGUUCCUUUAAGGG

siRNA#3：UCAUAUUCUGAAUCUCAUCCU

siRNA#4：UGUUAAAGAUGUAGUUCUAGU

siRNA#5：GGUAUGAAAUUGUUGUGUAUA

The pWPXL, pWPXL-LINC01139, pWPXL-shLINC01139 plasmid was co-transfected into cells along with the packaging plasmid ps-PAX2 and the envelope plasmid pMD2G using Lipofectamine 2000 (Invitrogen) as previously described. The virus particles were harvested 48 hours after co-transfection and then individually used to infect HCC cancer cells to generate corresponding stable cell lines. The efficiency of LINC01139 overexpression or knockdown was assessed by qRT-PCR.

**Cell proliferation assays**

For Cell Counting Kit-8 (CCK8) assay, MHCC97H and HCCLM3 cells（1×10^3^）were seeded into six pairs of duplicate wells of a 96-well plate and incubated at 37°C for 1–3 days. The old medium was removed, and then 100 µl fresh medium containing 1/10 volume of CCK-8 was added to wells at 24, 48, 72, 96, and 120h, respectively. Absorbance was detected daily after incubation at 37°C for 2h.

**Transwell invasion assays**

For invasion assays, MHCC97H and HCCLM3 cells (1×10^4^) in serum-free medium were added to the Transwell upper chamber, with the lower chamber containing 700 µl of medium with 10% FBS. After 48h at 37°C in an incubator at 5% CO_2_, the migrated cells present on the underside of the Transwell membrane were fixed with methanol and stained with crystal violet. Cell numbers were counted in five random fields.

***In vivo* assays**

The animal studies were performed in accordance with the institutional ethics guidelines for animal experiments approved by the animal management committee. Approximately 5×10^5^ Hep1-6 cells were suspended in 200μl serum-free DMEM medium and subcutaneously injected into the flank of each C57BL/6 mice (Female, 4–6-week-old). When the average tumour size reached approximately 60mm^3^, mice were evenly divided into control and treatment groups based on tumour volume. The mice in the treatment group were administered intraperitoneal injections of 100μg/100μl of anti-PD-L1 antibody. The treatment was administered twice a week for two consecutive weeks. Tumour measurements were performed every other day, and tumour volumes were calculated using the formula: V(mm^3^) = width^2^(mm^2^) × length(mm)/2. For ethical considerations, mice were sacrificed when the tumour volumes reached 1000mm^3^ and tumours were removed for further study.

**Collection of glucose metabolism-related genes**

All the glucose metabolism-related genes were collected from the Kyoto Encyclopedia of Genes and Genomes (KEGG) ^1^. We selected the glycolysis pathway named ‘Glycolysis/Gluconeogenesis’ for Homo sapiens. In total, 67 glucose metabolism-related genes were obtained in this study.

**Genetic and transcriptome profiles of HCC**

We collected the genetic profiles of HCC patients from cBioPortal ^2;3^. In total, 379 samples from the ‘Liver Hepatocellular Carcinoma (TCGA, Firehose Legacy)’ project were downloaded. We queried by the list of glucose metabolism-related genes to obtain all the genetic profiles of them, including somatic mutations and copy-number alterations.

In addition, the transcriptome profiles of HCC patients were downloaded using R package ‘TCGAbiolinks’ ^4^, including protein-coding genes and non-coding genes. In total, 377 samples with both genetic and transcriptome profiles were retained for our analysis. In addition, Genes that were not expressed in >50% samples were deleted from our analyses. In total, 6302 lncRNAs were included in subsequent analysis.

**Clinical characteristics of HCC**

The clinical information of HCC patients was also downloaded from cBioPortal, including sex, metastasis, tumor stage, and tumor mutation burden (TMB), hypoxia score, survival status, and survival days. Based the genetic alterations of glucose metabolism-related genes, we divided the patients into two groups, with genetic alteration or without genetic alteration of glucose metabolism-related genes. The clinical characteristics between HCC patients were compared between two groups by Fisher’s exact test and Wilcoxon’s rank sum test.

**Tumor microenvironment of HCC**

We collected immune-related features such as immune cell infiltration, immune checkpoint genes and immune pathways. Immune cell infiltration levels of HCC samples were calculated by CIBERSORT algorithm ^5^, QUANTISEQ ^6^, MCPCOUNTER ^7^ and xCell ^8^, which were collected from TIMER2.0 ^9^. In addition, 34 immune checkpoint genes were obtained from a recent study ^10^. In total, 50 cancer- and immune-related pathways used in this analysis were downloaded from MSigDB database ^11^. We calculated the enrichment score of the immune pathways for each HCC sample by the GSVA algorithm ^12^. Next, pathway enrichment scores between samples with or without genetic alteration of glucose metabolism-related genes were compared by Wilcoxon’s rank sum test.

**Prioritization of glucose metabolism-related lncRNAs**

Wilcoxon’s rank sum test was used to evaluate the expression differences of lncRNAs between HCC samples with or without genetic alteration of glucose metabolism-related genes. In addition, the fold change (FC) was used to evaluate the expression levels of genes or lncRNAs in different groups, which was calculated by dividing the average gene or lncRNA expression of samples with genetic alteration by the average gene or lncRNA expression of samples without genetic alteration. When FC>1, it indicated up-regulation in the genetic alteration group, while <1 indicated down regulation. False discovery rate (FDR) was used to adjust the p values, and genes or lncRNAs with FDR<0.05 were considered as differentially expressed.

We calculated the Spearman correlation coefficient between the enrichment scores of immune pathways and the expressions of 82 differentially expressed lncRNAs. The lncRNA-pathway pairs with p-value<0.05 was considered as significant correlated. Similar analysis for immune checkpoint genes and immune cell infiltration levels was performed. Next, we ranked the differentially expressed lncRNAs according to the number of correlated immune-related features.

**References**

1. Kanehisa M, Furumichi M, Sato Y, Kawashima M, Ishiguro-Watanabe M. KEGG for taxonomy-based analysis of pathways and genomes. *Nucleic Acids Res.* 2023;51(D1):D587-D592.

2. Gao J, Aksoy BA, Dogrusoz U, et al. Integrative analysis of complex cancer genomics and clinical profiles using the cBioPortal. *Sci Signal.* 2013;6(269):pl1.

3. Cerami E, Gao J, Dogrusoz U, et al. The cBio cancer genomics portal: an open platform for exploring multidimensional cancer genomics data. *Cancer Discov.* 2012;2(5):401-404.

4. Colaprico A, Silva TC, Olsen C, et al. TCGAbiolinks: an R/Bioconductor package for integrative analysis of TCGA data. *Nucleic Acids Res.* 2016;44(8):e71.

5. Newman AM, Liu CL, Green MR, et al. Robust enumeration of cell subsets from tissue expression profiles. *Nat Methods.* 2015;12(5):453-457.

6. Finotello F, Mayer C, Plattner C, et al. Molecular and pharmacological modulators of the tumor immune contexture revealed by deconvolution of RNA-seq data. *Genome Med.* 2019;11(1):34.

7. Becht E, Giraldo NA, Lacroix L, et al. Estimating the population abundance of tissue-infiltrating immune and stromal cell populations using gene expression. *Genome Biol.* 2016;17(1):218.

8. Aran D, Hu Z, Butte AJ. xCell: digitally portraying the tissue cellular heterogeneity landscape. *Genome Biol.* 2017;18(1):220.

9. Li T, Fu J, Zeng Z, et al. TIMER2.0 for analysis of tumor-infiltrating immune cells. *Nucleic Acids Res.* 2020;48(W1):W509-W514.

10. Sherif S, Roelands J, Mifsud W, et al. The immune landscape of solid pediatric tumors. *J Exp Clin Cancer Res.* 2022;41(1):199.

11. Liberzon A, Birger C, Thorvaldsdottir H, Ghandi M, Mesirov JP, Tamayo P. The Molecular Signatures Database (MSigDB) hallmark gene set collection. *Cell Syst.* 2015;1(6):417-425.

12. Hanzelmann S, Castelo R, Guinney J. GSVA: gene set variation analysis for microarray and RNA-seq data. *BMC Bioinformatics.* 2013;14:7.


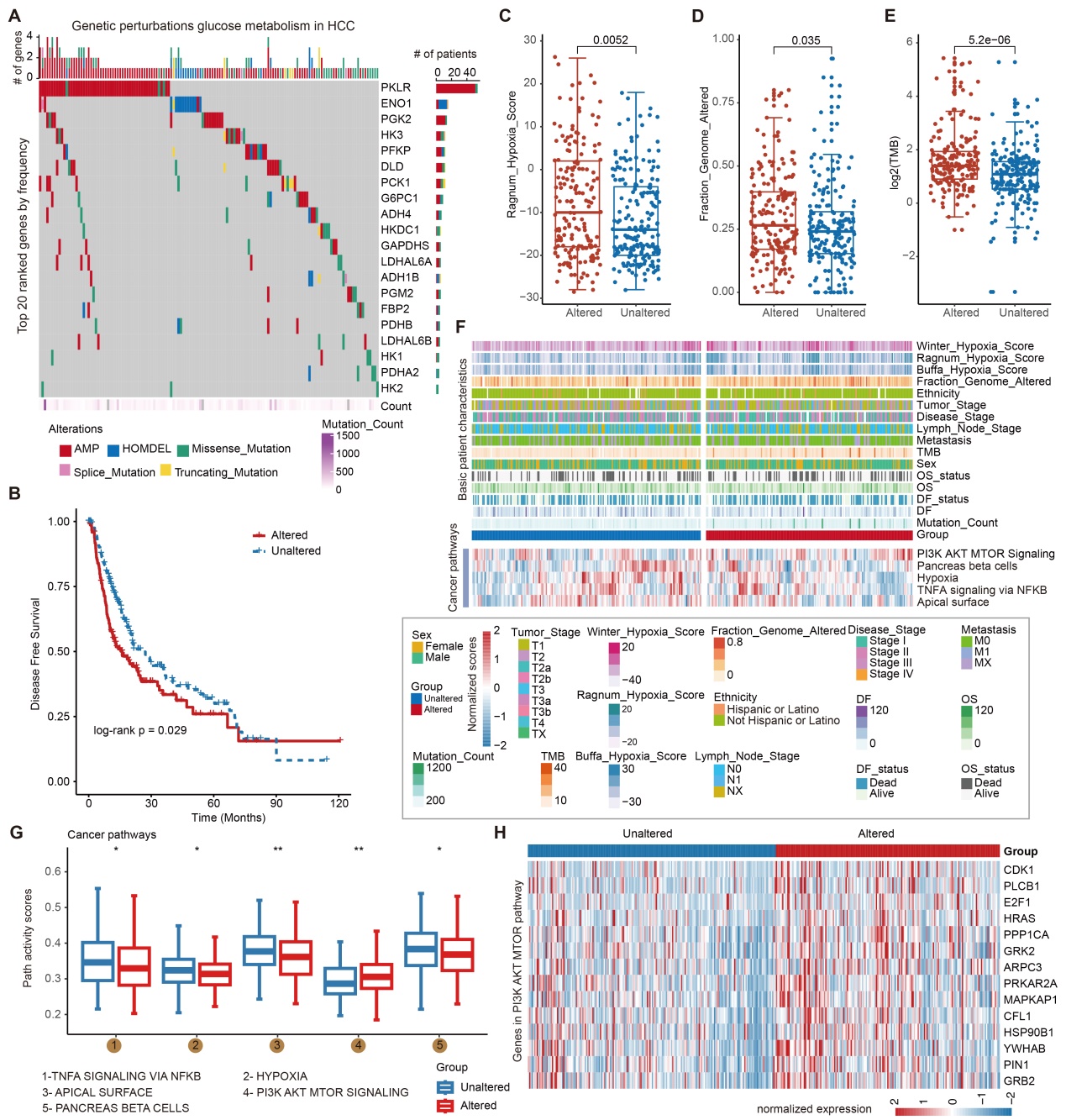


**Figure S1. Widespread genetic alterations of glucose metabolism-related genes correlated with perturbations of cancer and immune-related pathways.** A, Top ranked 20 genes by genetic alteration frequency. B, Kaplan-Meier disease-free survival curves. All patients were divided into two groups based on with or without glucose metabolism-related genes alterations. C-E, Boxplots showing the distributions of hypoxia scores, fraction of genome altered and TMB for two groups of patients. C for hypoxia scores, D for fraction of genome altered, E for TMB. F, Heat map showing the activities of cancer-related pathways and clinical features for HCC patients. G, Boxplot showing the activity scores of PI3K-AKT-MTOR pathway. H, heat map showing the expressions of genes involved in PI3K-AKT-MTOR pathway.


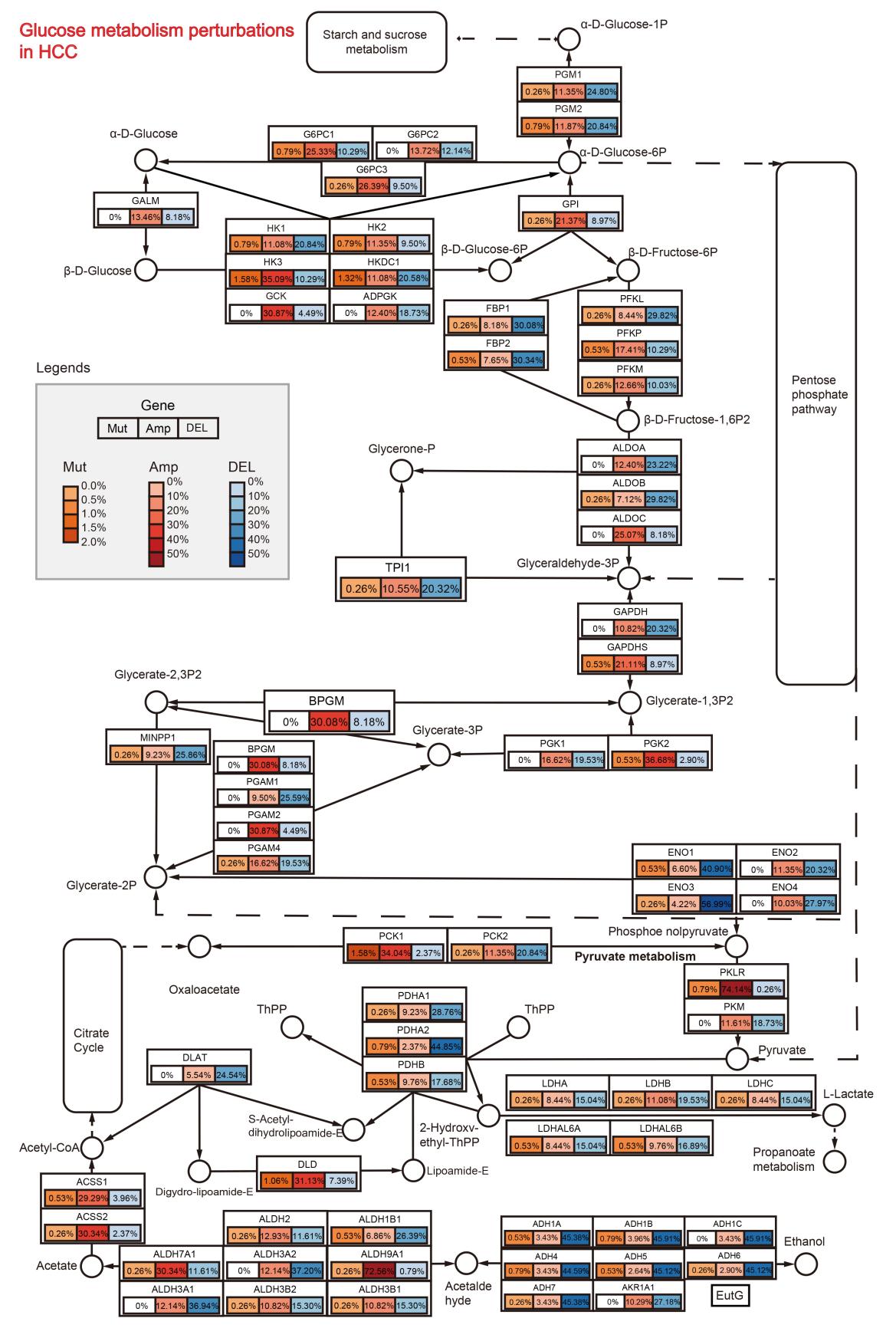


**Figure S2. Landscape of genetic alterations of glucose metabolism in HCC.** Each gene represents by three parts which indicated the frequency of genetic alterations, including mutation, copy number amplification and deletion.


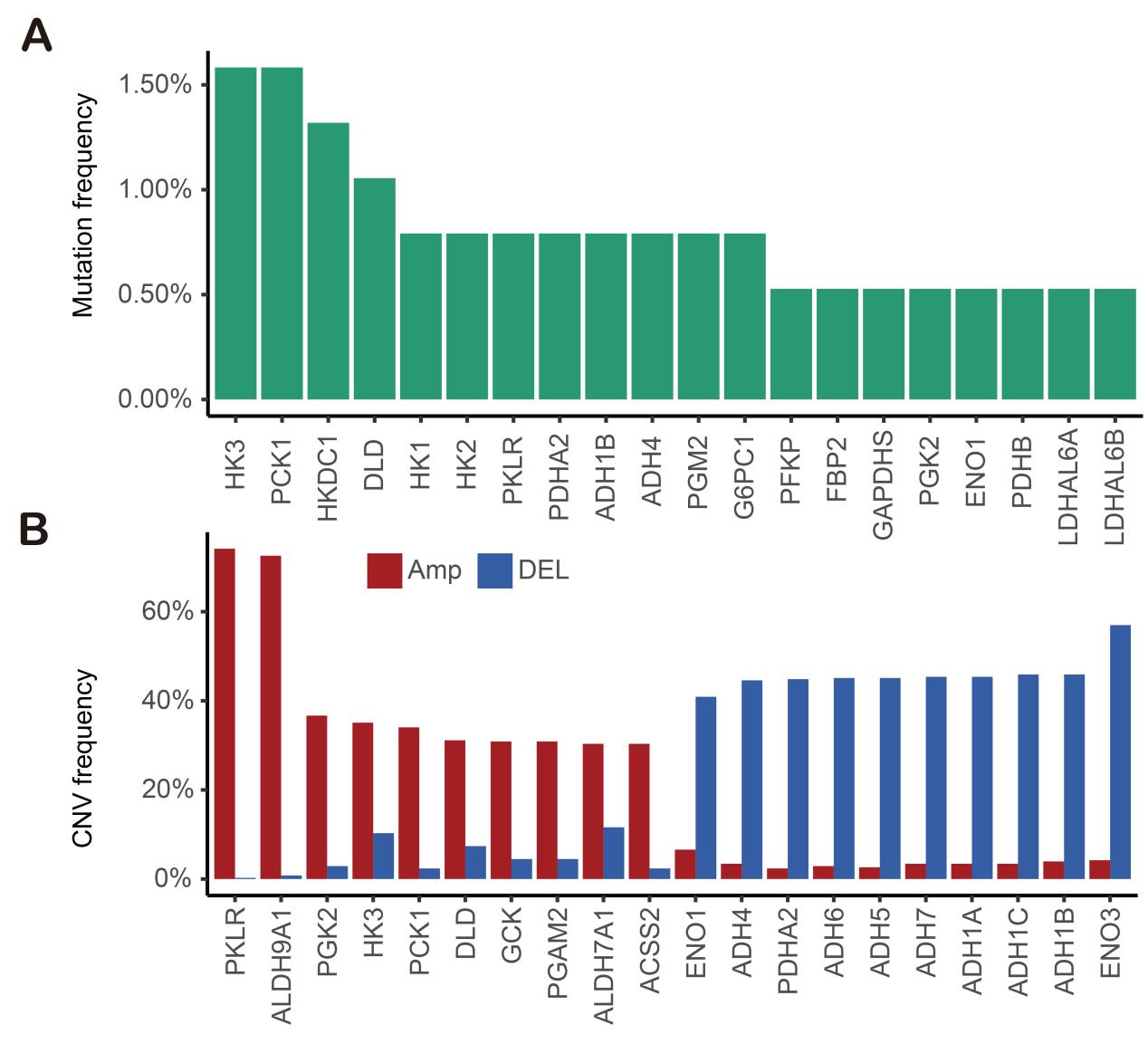


**Figure S3. Top ranked genes with high frequency of genomic alterations.** A, Top ranked genes with high mutation frequency. B, Top ranked genes with high CNV frequency.


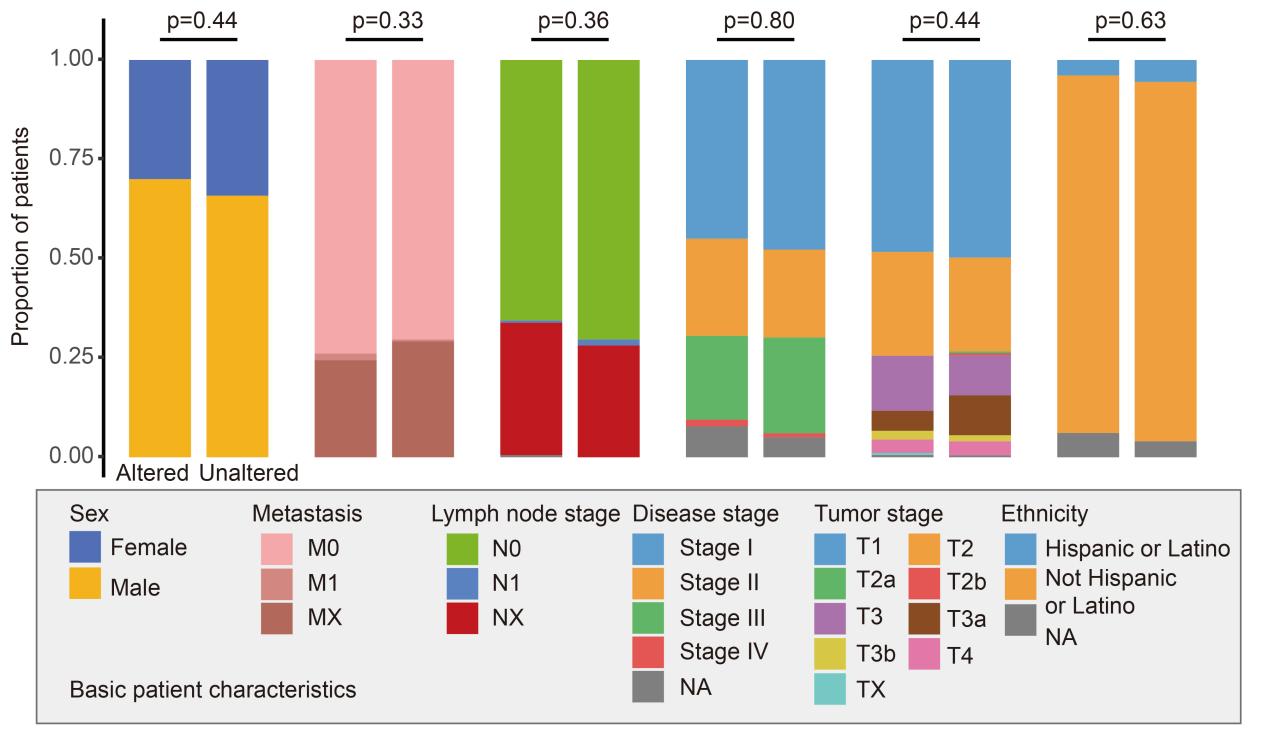


**Figure S4. Clinical significance of patients with vs. without glucose metabolism alterations in HCC.**


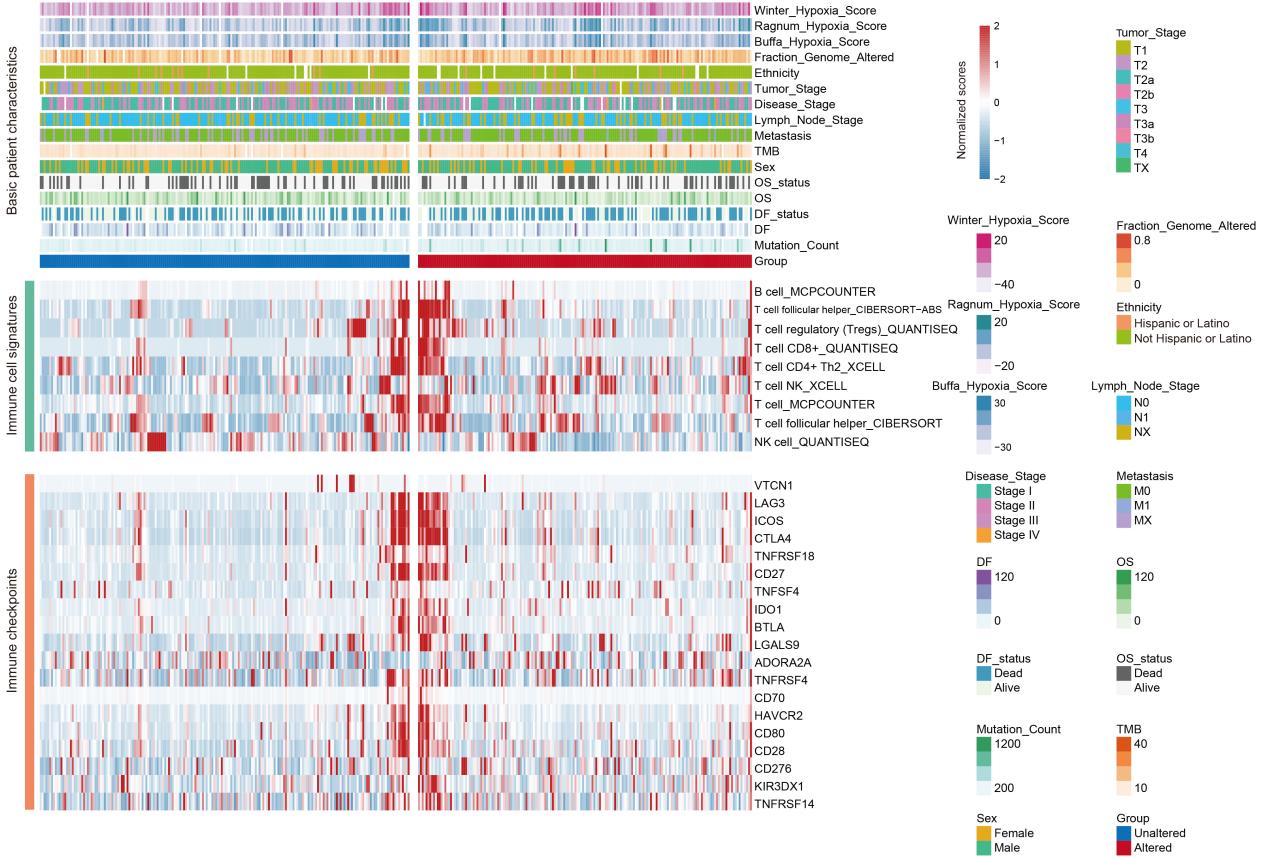


**Figure S5. Heat maps showing the activities of immune and cancer-related signatures in patients with vs. without glucose metabolism alterations in HCC.**


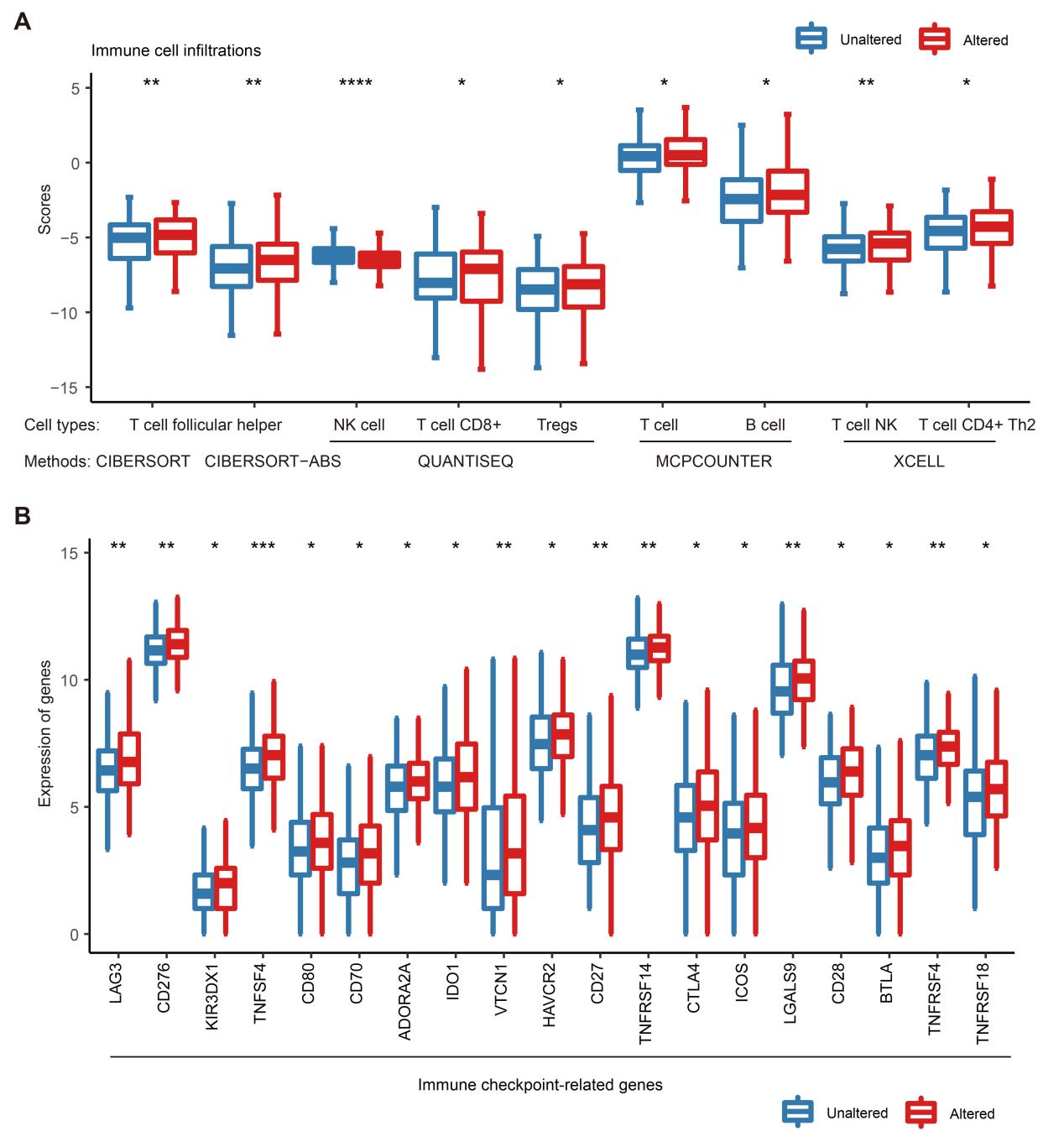


**Figure S6. Significant differences of immune cells infiltrations and expression of immune checkpoints in patients with vs. without glucose metabolism alterations.** A for immune cell infiltrations. B for expressions of immune checkpoints.


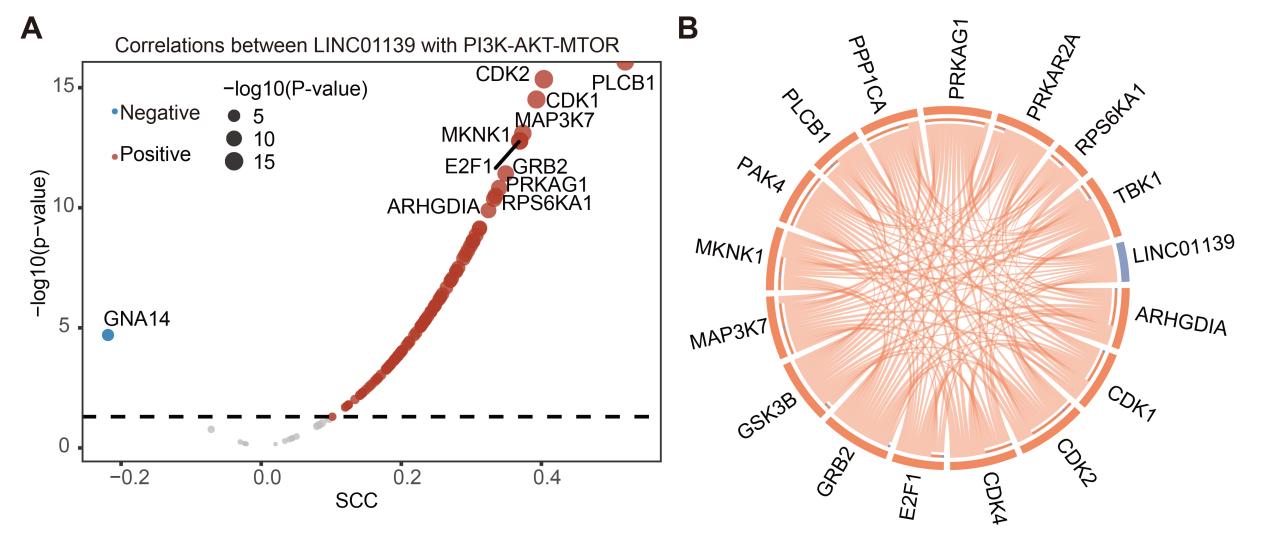


**Figure S7. Correlation of LINC01139 and PI3K-ATK-MTOR pathway.** A, Scatter plot showing the correlations between expressions of LINC01139 and genes in PI3K-ATK-MTOR pathway. B, Circos plot showing the genes correlated with LINC01139. Only genes with R>0.3 were shown.


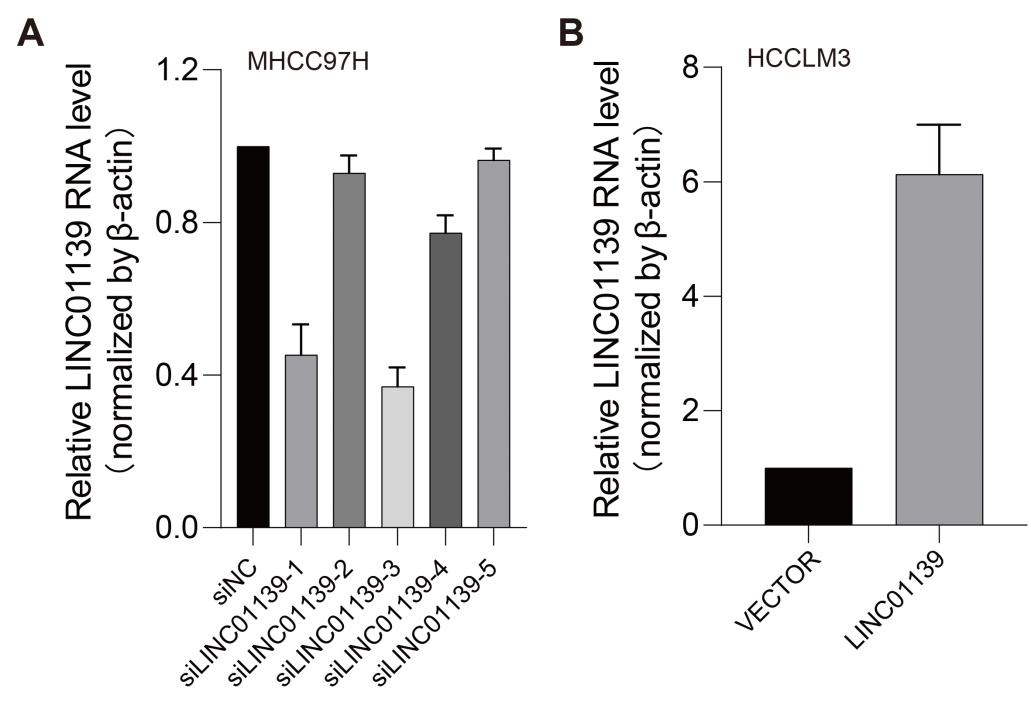


**Figure S8. Knockdown and overexpression of LINC01139 in HCC cell lines.** A, Relative expression of LINC01139 using five shRNAs. B, Relative expression of LINC01139 when overexpression.


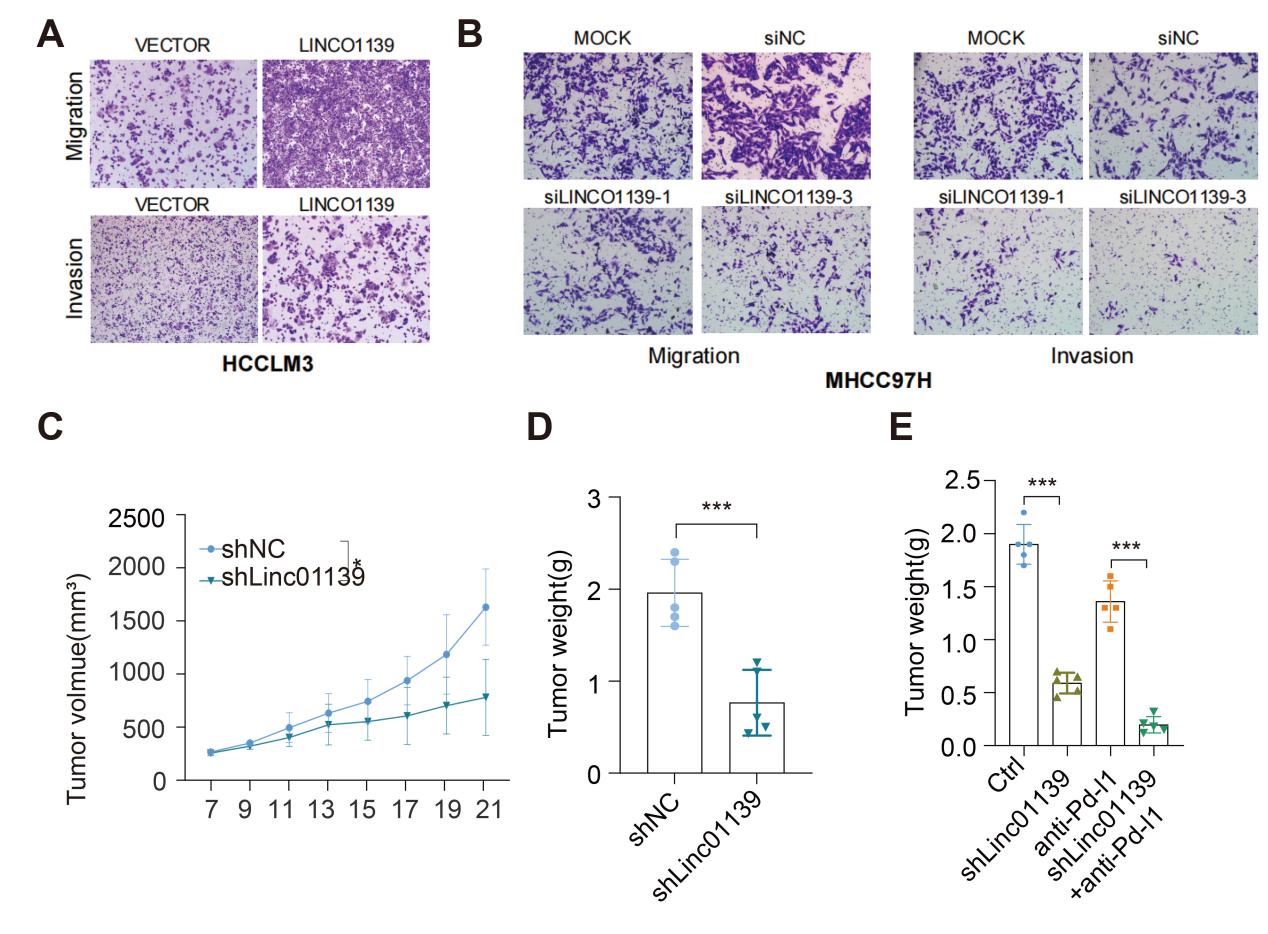


**Figure S9. Linc01139 promotes tumor growth, migration and invasion in HCC.** A, Representative images showing the effects of LINC01139 overexpression on migration and invasion. B, Representative images showing the effects of LINC01139 knockdown on migration and invasion. C, Line graph showing the effects of linc01139 knockdown on tumor volumes. D, Bar plot showing the effects of linc01139 knockdown on tumor weights. E, Tumor weights of mice treated with ctrl, shLinc01139, anti-Pd-l1 and anti-Pd-l1+shLin01139.
